# Teaching AI for Radiology Applications: a Multisociety-Recommended Syllabus from the AAPM, ACR, RSNA, and SIIM

**DOI:** 10.1007/s10278-025-01485-8

**Published:** 2025-10-01

**Authors:** Felipe Kitamura, Timothy Kline, Daniel Warren, Linda Moy, Roxana Daneshjou, Farhad Maleki, Igor Santos, Judy Gichoya, Walter Wiggins, Brian Bialecki, Kevin O’Donnell, Adam E. Flanders, Matt Morgan, Nabile Safdar, Katherine P. Andriole, Raym Geis, Bibb Allen, Keith Dreyer, Matt Lungren, Monica J. Wood, Marc Kohli, Steve Langer, George Shih, Eduardo Farina, Charles E. Kahn, Ingrid Reiser, Maryellen Giger, Christoph Wald, John Mongan, Tessa Cook, Neil Tenenholtz

**Affiliations:** 1 Society of Imaging Informatics in Medicine, Leesburg, USA; 2 Bunkerhill Health, San Francisco, CA USA; 3 Universidade Federal de São Paulo, São Paulo, Brazil; 4 Department of Radiology, Mayo Clinic, Rochester, MN USA; 5 Carle College of Medicine, University of Illinois Urbana-Champaign, Urbana, USA; 6 New York University Grossman School of Medicine, New York, NY USA; 7 Department of Biomedical Data Science and Dermatology, Stanford School of Medicine, Stanford, CA USA; 8 Department of Computer Science, University of Calgary, Calgary, Alberta Canada; 9 Ionic Health, Porto, Portugal; 10 Emory University, Atlanta, GA USA; 11 Greensboro Radiology, Durham, NC USA; 12 Department of Radiology, Duke University Health System, Durham, NC USA; 13 American College of Radiology, Reston, VA USA; 14 Canon Medical Research USA, Vernon Hills, USA; 15 Thomas Jefferson University, Philadelphia, PA USA; 16 University of Utah, Salt Lake City, UT USA; 17 Brigham and Women’s Hospital, Harvard Medical School, Mass General Brigham AI, Boston, MA USA; 18 National Jewish Health, Denver, CO USA; 19 Department of Radiology, University of Colorado School of Medicine, Aurora, CO USA; 20 Birmingham Radiological Group, Birmingham, AL USA; 21 American College of Radiology, Data Science Institute, Reston, VA USA; 22 Mass General Brigham, Boston, MA USA; 23 Department of Radiology and Biomedical Imaging, University of California San Francisco, San Francisco, CA USA; 24 Department of Biomedical Data Science, Stanford University, Stanford, CA USA; 25 Microsoft Health and Life Sciences, Mountain View, CA USA; 26 Mount Auburn Hospital, Harvard Medical School, Cambridge, MA USA; 27 Weill Cornell Medicine, New York, NY USA; 28 Dasa, São Paulo, Brazil; 29 University of Pennsylvania, Philadelphia, PA USA; 30 University of Chicago, Chicago, IL USA; 31 Radiological Society of North America, Oak Brook, IL USA; 32 Microsoft Research, Cambridge, MA USA

**Keywords:** AI Education, Radiology, Teaching

## Introduction

Medical imaging is undergoing a transformation driven by the advent of new, highly effective, machine learning techniques paired with increases in computational capabilities (Cheng et al. [3]; [1, 8, 12]). These advanced algorithms have the potential to improve disease detection, diagnosis, prognosis, and treatment outcomes. However, the complexity of machine learning models, the large amounts of curated and annotated data required by some methods, and the potential for bias and error make it challenging for individuals to safely and effectively leverage these methods [10, 13, 14, 25, 26].

To address these challenges, the American Association of Physicists in Medicine (AAPM), American College of Radiology (ACR), Radiological Society of North America (RSNA), and Society for Imaging Informatics in Medicine (SIIM) have worked together to develop a syllabus detailing a recommended set of competencies for medical imaging professionals interacting with these systems. This guide is aimed at four different personas: users of AI systems, purchasers of AI systems, individuals who provide clinical expertise during the development of AI systems (“clinical collaborators”), and developers of AI systems.^1^

This is a syllabus, not a curriculum, and is intentional in this scope. Recognizing that individuals may benefit from different presentations of the same material, this work enumerates a series of relevant competencies but does not prescribe, nor offer, a method of instruction (Schuur et al. [19]; [7]). By addressing the task-specific demands of each role, this guide will enable medical imaging professionals to utilize machine learning systems more safely and effectively, ultimately improving patient care and outcomes.

## User

While there’s a rich history of research seeking to improve patient care by leveraging AI [17], clinical deployment has often lagged due to the models’ limited capabilities. However, this has recently changed with modern advances in AI driven by increasingly powerful GPUs, larger internet-scale datasets, and more efficient open-source programming frameworks [2]. This has expanded the scope of potential users to include a broader set of individuals and organizations, such as physicians, residents, nurses, hospitals, clinics, payers, pharmaceutical companies, and government agencies, many of which may lack familiarity with these models’ strengths and weaknesses [20].

Users of AI should recognize the algorithms carry both powerful predictive capabilities as well as their distinctly non-human failure modes. The latter is especially vital to mitigate the risk of automation bias and other systemic errors. Users should also be aware of the intended use of the algorithm as cleared by the FDA—utilizing AI tools beyond their approved scope may lead to unexpected or inaccurate outcomes. It’s therefore recommended that users familiarize themselves with the FDA labeling to understand a tool’s cleared use case and potential limitations.

These systems are designed to provide a response representative of the training distribution. Therefore, any biases present in the training data may also be found in the model’s responses, and conversely, any inputs that vary dramatically from the training data may result in unexpected responses [5, 18]. This can occur with changes in the patient population, scanners, imaging protocols, or a variety of other scenarios. Users should be aware that sudden, adverse changes in model performance may occur and have a point-of-contact to alert should this arise.

Complicating matters, many AI algorithms are not designed to be explainable, meaning they may be unable to provide evidence as to how or why a given prediction was made. In such cases, pseudo-explanations may be derived (e.g., saliency maps) but may lack the reliability of models explicitly designed with explainability in mind [11]. To ensure patient safety, users should understand the differences between the two and recognize how to interpret them.

Table 1 summarizes the relevant material that would well-equip users of AI systems in imaging workflows. A more detailed version may be found in the appendix.

**Table 1:** Competencies beneficial to users of AI systems in imaging workflows

Properties of AI algorithms
Using AI algorithms effectively
Limitations of AI
Safe uses of AI
Importance of user feedback

## Purchaser

As the prevalence of AI deployments increases and the market for AI solutions becomes more rich, those responsible for these purchasing decisions will be more frequently evaluating algorithms for safety, efficacy, reliability, transparency, and value. Decisions will frequently involve balancing potential benefits, costs, and implementation risks. Benefits may range from improved patient outcomes to reductions in operating costs to opportunities for increased revenue. While some costs may be direct, e.g., acquisition and licensing fees, there may also be indirect costs that should be considered, including IT staff support, upgrade/maintenance fees, and potential hardware purchases. Additionally, some algorithms, while beneficial for patient outcomes, may not yield a positive return on investment for the institution. Thus, buyers should carefully weigh an algorithm's benefits, across all axes, against the existing standard of care and other alternatives [16].

While metrics such as accuracy, sensitivity, and specificity are common means of performance evaluation, the purchaser may also wish to consult FDA labeling to consider the population over which the metrics were calculated to ensure suitability for the proposed workflow. This can include—among other things—dataset diversity, population characteristics, and exclusion criteria, which may or may not be consistent with the deployment environment. In some instances, this information may not be readily available. The ACR Data Science Institute has been soliciting descriptive data elements from AI manufacturers as part of the AI Transparency effort launched at RSNA 2023 [22]. This ongoing process is designed to better inform purchasing and deployment decisions by stakeholders. The data elements and other relevant information, such as 510 K summaries and instructions for use documents, are freely available online^2^ and are continually being updated.

Beyond measures of algorithmic accuracy, a successful deployment requires the execution of model inference, format of model output, and consumption of results be compatible with the envisioned workflow. Compute should be provisioned to permit the desired inference volume to be executed within the specified latency (the time required to make a prediction) bounds to ensure other parts of the workflow are not delayed. Results should also be presented to the user in a location and format fit for consumption, allowing them to act on the results with minimal friction. Finally, once integrated, an assessment may be performed to quantify the realized return on investment.

The purchaser’s responsibilities extend beyond the initial acquisition. Policies and procedures should be established for the implementation, ongoing monitoring, and continual evaluation of the algorithm to detect any potential degradation in accuracy or adverse effects. The purchaser should be aware that these capabilities incur additional costs/resources and thus should be budgeted for in any cost estimation.

Throughout the model acquisition and deployment lifecycle, purchasers may also benefit from the input of a governance committee that includes representatives from the diverse set of stakeholders within the institution or health system. This may include, among others, healthcare providers, technologists, informaticians, and IT staff [4]. When present, a governance framework should be established to ensure a clear assignment of responsibilities. This encourages stakeholder input on proposed changes as well as progress on relevant action items.

Table 2 summarizes the relevant material that would well-equip purchasers of AI systems for imaging workflows. A more detailed version may be found in the appendix.

**Table 2:** Competencies beneficial to purchasers of AI systems for imaging workflows

Pre-engagement product/vendor evaluation
Pre-deployment site compatibility/suitability evaluation
Pre-deployment performance evaluation
Post-deployment monitoring/evaluation
Model value assessment

## Physician Collaborator

When developing AI algorithms for imaging applications, clinical expertise is required to inform the use case and interpret the available data. Physician Collaborators therefore play an important role in all stages of the development process, including use case selection, dataset curation, data interpretation, model creation, and algorithm testing and monitoring [24].

To ensure efficient investment of resources, both by the developer and potential purchasers, use cases should be formulated and refined to maximize utility. Physician Collaborators may leverage their expertise in clinical workflows and data generation processes to inform use case definition, which includes a precise task description as well as identifying the intended user and the environment in which they operate [15]. For clinical workflows, the Physician Collaborator may also provide feedback on the format of the model output to maximize its utility and assist in the definition of task-specific metrics that best quantify delivered value.

The Physician Collaborator will also often be consulted during dataset curation. Knowledge of the data selection and preparation processes is therefore highly beneficial. Leveraging their clinical competencies, they may help define labeling schemes, annotation techniques, and the reference standards that are most appropriate for the use case. This may take the form of communicating label fidelity/variability as well as any biases that might exist in the data, neither of which may be immediately apparent to a non-expert. Together with the other members of the development team, their collective expertise can be utilized to mitigate the resulting risks.

In addition to dataset curation, the Physician Collaborator may also be asked to help evaluate and provide context to a model’s performance. Such requests may occur at any point in the model development lifecycle, ranging from metric definition in the earliest stages to comparisons of a release candidate against an unaided user or workflow. Familiarity with common performance metrics and an understanding of their differences can therefore be beneficial in these scenarios [6]. Combined with clinical acumen, correlations between quantitative performance metrics and improvements in workflow can be determined and help inform the development roadmap.

Beyond raw performance measures, the Physician Collaborator may be consulted to provide deeper context on failure modes (e.g., uncorrelated errors vs mimics) as well as their broader implications (e.g., the impact on the delivery of care or patient wellbeing). One particularly common yet consequential example is identifying and correcting potential biases that may be impacting model performance. While algorithm developers may be able to identify these errors without a collaborator’s assistance, they often lack the detailed knowledge of care pathways to determine their source and formulate mitigation strategies.

Finally, once an initial version of an algorithm is developed, the Physician Collaborator may be asked to serve as one of its earliest users. Leveraging their medical training, knowledge of the clinical workflow in a variety of environments, and understanding of the model’s design, the Physician Collaborator is uniquely positioned in these circumstances to provide holistic feedback on model performance and identify pain points in the workflow. This can often produce valuable insights into areas for model improvement as well as their urgency.

Table 3 summarizes the relevant material that would well-equip physicians collaborating in the development of AI systems for clinical workflows. A more detailed version may be found in the appendix.

**Table 3:** Competencies beneficial to physician collaborators in AI model development

Use case definition
Dataset curation
Model evaluation
Alpha testing early clinical implementations

## Developer

In addition to the knowledge required to develop an AI algorithm in non-clinical domains,^3^ developers of AI in healthcare should also be aware of the intricacies of clinical data and delivery of care. A fundamental component of this is the users, their workflows, and the environments in which they operate—all of which may be radically different from those encountered in other domains. Unlike many applications where the impact of an incorrect prediction may be transient or inconsequential, inaccuracies in healthcare algorithms may result in more serious outcomes, including permanent harm to the patient or even death. Thus, regular communication with physician collaborators and/or users is vital to bridge these potential gaps in knowledge and mitigate potential risks.

Early in the development process, this communication can facilitate the identification of high-leverage use cases, namely bottlenecks in imaging workflows and pain points in the delivery of care. In addition to improving the interpretation accuracy of users, developers may also wish to consider how proposed applications will fit into existing workflows as well as the IT infrastructure and data necessary to implement them. This understanding can only come from deep familiarity with the clinical environment, thus encouraging communication with other key stakeholders.

Familiarity with the data acquisition processes, their formats, and transfer protocols is also beneficial. Healthcare-specific data formats often carry additional metadata which may be leveraged to improve predictive accuracy. Additionally, when communicating with other systems in the healthcare ecosystem, these standards are the transport protocols employed making them relevant for integration.

Beyond the simple communication with other systems, developers would also benefit from an understanding of how their model output is consumed in existing workflows. To maximally accelerate workflows and encourage adoption, model output should be presented in a relevant format and location. These details can be nuanced and/or subtle, further encouraging communication with other stakeholders [23].

Being a highly regulated domain, developers should also familiarize themselves with policies and procedures that regulate the marketing, sale, and deployment of clinical AI systems. With many falling under the umbrella of software-as-a-medical-device (SaMD), particular claims and use cases will require regulatory submission and approval (e.g., FDA 510(k) and Premarket Approval) prior to marketing. Additionally, due to the sensitivity of healthcare data, many design considerations must be followed to ensure compliance with local law (e.g., HIPAA and GDPR,). The FDA has released a manual of best practices covering many of these topics [9].

Finally, even once an algorithm is developed and has received regulatory approval, deployment may still prove to be challenging due to the nascency of deployment standards, clinical security systems, hardware limitations, and difficulties in logging and monitoring. As standards and accepted best practices are still emerging [21], these systems and processes may vary from site to site, introducing heterogeneity and complexity in deployment environments.

Table 4 summarizes the relevant material that would well-equip developers of AI systems for clinical workflows. A more detailed version may be found in the appendix.

**Table 4:** Competencies beneficial to developers of AI systems for clinical workflows

Problem Identification
Understanding medical data
Model evaluation/utility analysis
Algorithm deployment
AI regulation

## Conclusion

The integration of AI in imaging workflows is rapidly accelerating, which promises to profoundly transform clinical workflows with potential increases in efficiency and improvements in quality. However, for these gains to be realized, stakeholders should possess the requisite knowledge to safely and effectively develop, deploy, and leverage these systems.

We, the authors of this work representing the AAPM, ACR, RSNA, and SIIM, have therefore designed a syllabus that carries the breadth and depth to fulfill this criteria. We hope that this syllabus provides a strong foundation for didactic materials and a brighter future for medical imaging.

## Appendix

### AI Syllabus for Radiology Applications

#### User—Interacts with AI Algorithms in Their Clinical Workflow

- Properties of AI algorithms
  - Complexity, nonlinearity, and frequent opacity of AI algorithms
  - Algorithm outputs that mirror the training distribution
- Using AI algorithms effectively
  - Identification of approved regulatory claims and the implications of supplying data from alternative workflows
  - Differentiating between model predictions and information derived from these predictions
  - Evaluating AI output in the context of patient history
- Limitations of AI
  - Lack transparency in their predictions
  - Inheritance of biases from training datasets
  - Unpredictable, nonhuman failure modes
  - Potential brittleness with out-of-distribution data, including unseen scanners, protocols, and patient populations
- Safe use of AI
  - Inclusion criteria for algorithm use
  - Identification of failure modes
  - Risks of workflow modification
  - Risks of automation bias and methods of mitigation
- Importance of user feedback
  - Communication of feedback on algorithm performance
  - Identification of opportunities for improvement
  - Participation in initiatives (e.g., national registries) to share experiences and insightsurchaser/Clinical Owner—Makes

#### Purchaser / Clinical Owner– Makes purchasing decisions and is responsible for deployed algorithm﻿s

- Pre-engagement Product / Vendor Evaluation
  - Metrics of assessment (including measures of bias)
  - Review of data used for training and evaluation
  - FDA AI/ML Action Plan
- Pre-deployment Site Compatibility / Suitability Evaluation
  - Integration into the clinical workflow (standard vs bespoke integration)
  - Interoperability of the AI software and associated standards
  - Need for explainability or interpretability of AI results
  - Platform requirements
    - Hardware requirements (CPU, GPU, memory, storage)
    - On-premise vs cloud deployments
  - Data security / privacy
  - Data Use Agreements
  - Training users in appropriate use
  - Established definition of errors and subjective difference in opinion
- Pre-deployment Performance Evaluation
  - Testing with local datasets for early detection of failure modes
  - Shadow/canary deployment with acceptance criteria
  - Evaluation on relevant patient population and clinical environment
  - Detection of potential biases in observed results
- Post-deployment Monitoring / Evaluation
  - Collection and evaluation of user feedback
  - Sources of data drift and their impact
    - Scanners
    - Protocols
    - Patient Population
    - Collection process
  - Concept drift
  - Monitoring to ensure appropriate access and detect potential intrusions
  - Execution of backup strategy as part of a broader disaster recovery plan
- Model Value Assessment
  - Vendor billing models
  - Provider billing models
  - Quantitative measures of improvements in efficiency
  - Alternate revenue streams / discounts

#### Physician Collaborator -- Works alongside model developers to define and evaluate algorithms

- Use Case Definition
  - Evaluation of the proposed task for relevance and value
  - Assessment of model output for ease of user consumption
  - Identification and communication of workflow nuances, especially if they may introduce bias
  - Identification of risks of off-label use
- Dataset Curation
  - Evaluation of tradeoffs in annotation techniques and label types
  - Assessment and communication of data quality
    - Label fidelity
    - Inter-rater variability
    - Potential for hidden biases
- Model Evaluation
  - Performance metrics
  - Communication of user experience and potential improvements
  - Identification of different types of failure modes (e.g., uncorrelated errors, mimics, etc.) and their implications
  - Bias / fairness evaluation
- Alpha-testing of early clinical implementations

#### Developer – Creates new AI algorithms to improve patient care and/or clinical workflows

- Problem Identification
  - Knowledge of the clinical imaging workflow (from ordering of imaging to impact of interpretation on treatment)
  - Identification of clinical pain points
  - Clinical IT infrastructure, environment, and imaging volume
  - Potential integration methods in existing workflows and their tradeoffs
    - Introduction of novel workflows
    - Augmentation of existing workflows
    - Replacement of existing workflows
- Understanding the Data
  - Medical imaging formats and transfer protocols (e.g., DICOM, HL7, FHIR)
  - Physical interpretation of pixel/voxel intensities in different modalities and/or series
  - Significance of pixel/voxel size, anisotropy, acquisition parameters, and other imaging properties
  - Privacy and data safety requirements (e.g., HIPAA, GDPR, etc.)
- Model Evaluation / Utility Analysis
  - Asymmetric costs of different error types
  - Clinical evaluation of ROI
  - Relevance of interpretability / explainability
- Algorithm Deployment
  - Nascent deployment standards
  - Hardware availability on-premise and in the cloud
  - Methods of logging and monitoring
  - Security requirements
- AI Regulation
  - Requirements of the relevant regulatory bodies (FDA, CE, etc.)
  - Differences between the various classes of risk / device classes
  - Limitations of regulatory approval/clearance

## Footnotes

1 In instances where individuals serve multiple roles, it is recommended they demonstrate competency in the material relevant to all of their expected responsibilities.

2 www.aicentral.org

3 This section assumes expertise in AI algorithm development, which is out-of-scope for this syllabus. It focuses instead on healthcare-specific competencies that a Developer new to healthcare may not possess.
